# Super selective percutaneous transhepatic coil embolization of intrahepatic pseudoaneurysm after pediatric liver transplantation: a case report

**DOI:** 10.1186/s42155-021-00221-x

**Published:** 2021-03-19

**Authors:** V. I. Huf, D. Grothues, B. Knoppke, H. Goessmann, W. A. Wohlgemuth, M. Melter, S. M. Brunner, H. J. Schlitt, W. Uller

**Affiliations:** 1 Department of Radiology, University Hospital Regensburg, Franz-Josef-Strauss-Allee 11, 93053 Regensburg, Germany; 2 KUNO University Children’s Hospital, University Hospital Regensburg, Franz-Josef-Strauss-Allee 11, 93053 Regensburg, Germany; 3 Department of Radiology, University Hospital Halle, Ernst-Grube-Str. 40, 06120 Halle, Germany; 4 Department of Surgery, University Hospital Regensburg, Franz-Josef-Strauss-Allee 11, 93053 Regensburg, Germany; 5 Faculty of Medicine, Department of Diagnostic and Interventional Radiology, Medical Center - University of Freiburg, University of Freiburg, Hugstetter Straße 55, 79106 Freiburg, Germany

**Keywords:** Pediatric liver transplantation, Percutaneous embolization, Pseudoaneurysm

## Abstract

**Background:**

Intrahepatic arterial pseudoaneurysms are a rare, life-threatening complication after pediatric liver transplantation. Treatment of choice represents interventional radiological management with endovascular embolization of the segmental artery proximal and distal to the aneurysm. However, this technique results in loss of arterial perfusion distal to the aneurysm with subsegment arterial ischemia.

**Case presentation:**

We report a case of a 1-year-old girl with a pseudoaneurysm in the split-liver graft. Direct percutaneous, transhepatic access to the pseudoaneurysm was performed followed by super selective coil application into the aneurysm.

**Conclusion:**

Super selective percutaneous, transhepatic coil application is feasible even in pediatric patients after liver transplantation and results in preservation of the entire course of the liver artery.

## Introduction

Pseudoaneurysms of the hepatic artery occur in 0.3–2.6% of cases after liver transplantation (St Michel et al. 2019). They may either develop spontaneously in the early as well as the late postoperative period or as iatrogenic complication after interventional biliary procedures (St Michel et al. 2019; Tessier et al. 2003). Clinically they may present with bleeding or elevated liver function tests. The mortality rate can be as high as 75%, which demonstrates the severity of such lesions (St Michel et al. 2019). Treatment of choice represents interventional radiological management with endovascular embolization of the segmental artery proximal and distal to the aneurysm, but this technique results in loss of arterial perfusion distal to the aneurysm with subsegment arterial ischemia. We report on direct percutaneous, transhepatic access to the pseudoaneurysm followed by super selective coil application into the aneurysm, in order to preserve the entire course of the liver artery.

## Case presentation

Living donor liver transplantation was performed with a left lateral split (Segment II / III) for biliary atresia at the age of 7 months. Three months after liver transplantation the patient experienced stenosis of the hepaticojejunostomy that was treated with percutaneous transhepatic biliary drain placement. An initially placed 6 french biliary drain was gradually upsized to 10 french. By the age of 13 months (body weight: 10.9 kg) the drain showed slightly bloody bile fluid. Immediately performed ultrasound and magnetic resonance imaging (MRI) diagnosed an intrahepatic pseudoaneurysm (5 × 6 × 7 mm) located within the central aspects of segment III artery. MRI was executed for treatment planning and to rule out additional vascular and non-vascular pathologies.

**Fig. 1:**
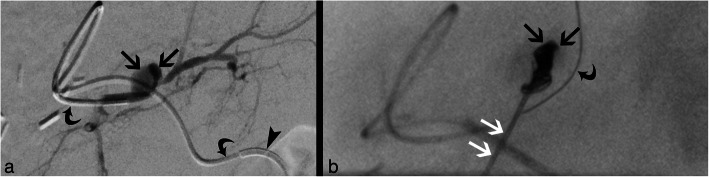
Percutaneous transhepatic coil embolization. **a** Angiogram of the segment III liver artery using the coaxial technique (macrocatheter: arrowhead; microcatheter: curved arrow) prior to embolization shows localization and configuration of pseudoaneurysm (black arrows) as well as irregularity of the segment III liver artery due to vasospasm. **b** Fluoroscopy after percutaneous transhepatic access and embolization of the pseudoaneurysm (black arrow) via the CHIBA needle (white arrows) delineates coils within the pseudoaneurysm. The microcatheter (curved arrow) was advanced into a side branch of S III artery distal to the pseudoaneurysm to avoid coil dislodgement

**Fig. 2:**
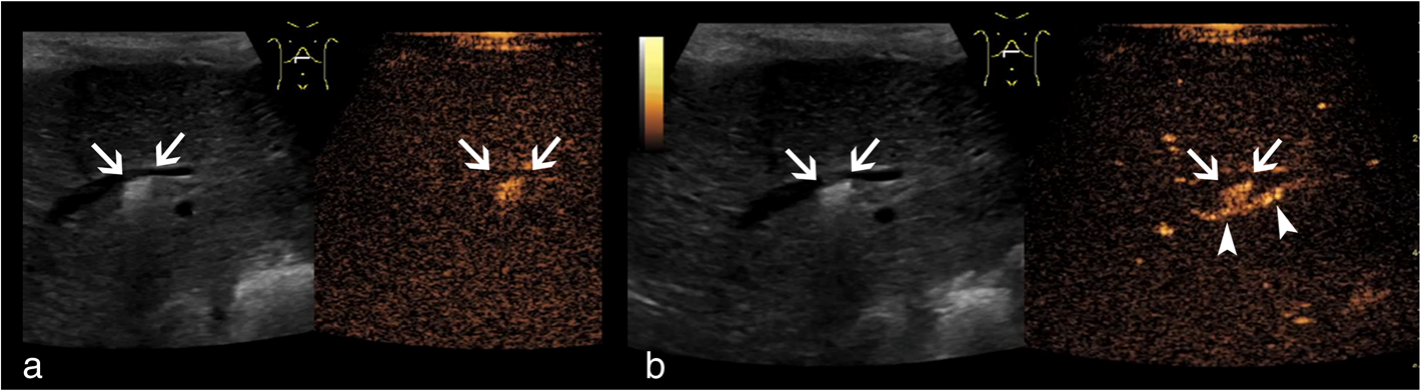
Post-interventional sonography displayed in contrast mode with split-screen; B-mode images are visible on the left, and contrast-mode images on the right side respectively. **a** Post-interventional ultrasound before contrast medium injection detects artifacts of coils (arrows) on B-mode (left side) contrast-mode images (right side). **b** Post-interventional contrast-enhanced sonography during arterial phase shows coils artifacts without contrast-enhancement of the pseudoaneurysm (arrow) and patency of the SIII artery (arrow heads) on B-mode (left side) and contrast-mode imaging (right side)

After removal of the biliary drain three attempts of percutaneous ultrasound-guided thrombin injections failed to occlude the pseudoaneurysm permanently. Consequently, coiling of the aneurysm was scheduled:

The right femoral artery was accessed and 100 IE heparin per kilogram body weight were administered. A microcatheter was placed in segment III artery using the coaxial technique (4F-RIM, Cordis, USA and Progreat, Terumo, Japan). The attempt to access the pseudoaneurysm with an endovascular placed catheter failed, due to anatomy and narrowing of the pseudoaneurysm’s neck and resulted in vasospasm of the segment III artery. Hence, the decision was made to access the pseudoaneurysm via a percutaneous, transhepatic approach. The microcatheter was advanced distal to the pseudoaneurysm to prevent coil dislodgement into the segmental artery and to facilitate coiling of the segmental artery proximal and distal to the pseudoaneurysm in case of rupture. Using ultrasound guidance, the pseudoaneurysm was successfully percutaneously punctured with a 22-gauche needle (CHIBA, Cook Medical, USA) and was completely filled with pushable coils (4 × 4 mm/2 mm, 4 × 3 mm/2 mm; Tornado, Cook, USA) (Fig. 1). Post-embolization angiography showed severe vasospasm of the segment III artery and 5 μg of nitroglycerine were injected twice via the microcatheter. Finally, vasospasm resolved by withdrawing the catheters (detected by ultrasound). Color-coded duplex sonography as well as contrast-enhanced ultrasound (CEUS) showed complete embolization of the pseudoaneurysm with preserved patency of the entire course of the segment artery immediately, 1, 2, 6 weeks and 3 months after embolization (Fig. 2). The parenchymal and biliary structures did not show any signs of ischemia.

## Discussion

Pseudoaneurysms of the intrahepatic arterial branches are a well-known complication after interventional biliary procedures (Tessier et al. 2003). In adults, treatment options depend on the pseudoaneurysms’ location, configuration, and size and include endovascular embolization, stent placement, or surgical ligation (St Michel et al. 2019). To date, a single description was published of percutaneous transhepatic coil embolization in an adult with the pseudoaneurysm measuring 1.5 cm (Millonig et al. 2004). However, very little is known about treatment of small arterial pseudoaneurysms in infants after split-liver transplantation.

Because of the very small diameter of segmental arteries and the pseudoaneurysms, as well as localization and configuration of pseudoaneurysms in pediatric split-liver transplants, surgery, stent placement, endovascular coiling of the aneurysm, and embolization of the segmental artery may be associated with an unfavorable risk-benefit profile resulting in loss of arterial perfusion and high morbidity. Ultrasound-guided thrombin injection has been reported as a treatment option for the occlusion of a pseudoaneurysm after liver laceration in an infant. However, this technique failed to occlude the aneurysm permanently in our case (Lorenz et al. 2013).

Direct percutaneous access and highly selective, exclusive embolization of the aneurysm prevents complications related to open surgery and even related to endovascular approaches.

We favored CEUS for follow-up since this technique is able to generate real time imaging without sedation and radiation exposure. Moreover, this technique has already proved to be a safe imaging technique in children after liver transplantation (Torres et al. 2019) and in the detection of pseudoaneurysms (Durkin et al. 2016).

## Conclusions

Super selective percutaneous, transhepatic coil application for treatment of intrahepatic pseudoaneurysm after split liver transplantation is feasible even in pediatric patients and CEUS proved to be an adequate tool for follow-up. Although, this reported minimally invasive management of a pseudoaneurysm after pediatric split liver transplantation requires an experienced multidisciplinary team, we think that this technique should be considered especially in pediatric patients to preserve arterial perfusion, to minimize graft ischemia, and consequently to reduce morbidity.

## Data Availability

Not applicable.

## References

[CR1] Durkin N, Deganello A, Sellars ME, Sidhu PS, Davenport M, Makin E (2016). Post-traumatic liver and splenic pseudoaneurysms in children: diagnosis, management, and follow-up screening using contrast enhanced ultrasound (CEUS). J Pediatr Surg.

[CR2] Lorenz JM, van Beek D, Van Ha TG, Lai J, Funaki B (2013). Percutaneous thrombin injection in an infant to treat hepatic artery pseudoaneurysm after failed embolization. Pediatr Radiol.

[CR3] Millonig G, Graziadei IW, Waldenberger P, Koenigsrainer A, Jaschke W, Vogel W (2004). Percutaneous management of a hepatic artery aneurysm: bleeding after liver transplantation. Cardiovasc Intervent Radiol.

[CR4] St Michel DP, Goussous N, Orr NL, Barth RN, Gray SH, LaMattina JC, Bruno DA (2019). Hepatic artery pseudoaneurysm in the liver transplant recipient: a case series. Case Rep Transplant.

[CR5] Tessier DJ, Fowl RJ, Stone WM, McKusick MA, Abbas MA, Sarr MG, Nagorney DM (2003). Iatrogenic hepatic artery pseudoaneurysms: an uncommon complication after hepatic, biliary, and pancreatic procedures. Ann Vasc Surg.

[CR6] Torres A, Koskinen SK, Gjertsen H, Fischler B (2019). Contrast-enhanced ultrasound for identifying circulatory complications after liver transplants in children. Pediatr Transplant.

